# Anterior tibial translation versus rotational instability in ACL reconstruction: Defining the problem before choosing the procedure

**DOI:** 10.1002/jeo2.70792

**Published:** 2026-05-29

**Authors:** Tomas Pineda, David H. Dejour, Volker Musahl, Romain Seil, Stefano Zaffagnini, Matthieu Ollivier

**Affiliations:** ^1^ Facultad de Medicina, Hospital del Trabajador Universidad Andrés Bello Santiago Chile; ^2^ Facultad de Medicina, Hospital el Carmen Universidad Finis Terrae Santiago Chile; ^3^ Orthopedic Surgery Department Lyon Ortho Clinic, Clinique de la Sauvegarde Lyon France; ^4^ Blue Cross of Western Pennsylvania Professor and Chief Sports Medicine, UPMC Freddie Fu Sports Medicine Center University of Pittsburgh Pittsburgh Pennsylvania USA; ^5^ Department of Orthopaedic Surgery Center Hospitalier de Luxembourg Luxembourg City Luxembourg; ^6^ Clinica Ortopedica e Traumatologica 2 IRCCS Istituto Ortopedico Rizzoli Bologna Italy; ^7^ Assistance Publique Hôpitaux de Marseille Institute for Locomotion Marseille France; ^8^ Département of Biomechanics, CNRS, ISM Aix‐Marseille University Marseille France

**Keywords:** anterior cruciate ligament reconstruction, instability phenotypes, posterior tibial slope, rotational instability, tibial slope–reducing osteotomy

**Abbreviations**
ACL: anterior cruciate ligament
LEAPs: lateral extra‐articular procedures
sATT: static anterior tibial translation
TDO: tibial deflexion osteotomy

The expanding use of lateral extra‐articular procedures (LEAPs) and tibial deflexion osteotomy (TDO)—also referred to as slope‐reducing anterior closing wedge high tibial osteotomy—in anterior cruciate ligament (ACL) reconstruction reflects meaningful progress in our understanding of mechanisms of graft failure. However, as their use expands, an important distinction must be maintained: they do not address the same problem.

## DIFFERENT BIOMECHANICAL PROBLEMS, DIFFERENT SURGICAL TOOLS

**Figure 1:**
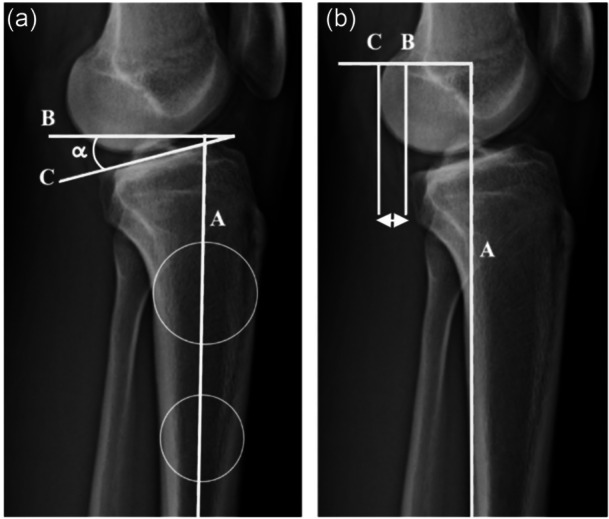
Radiographic assessment of sagittal‐plane parameters on lateral knee radiographs. (a) Measurement of posterior tibial slope, defined as angle *α* between the medial tibial plateau and the anatomical axis of the proximal tibia. (b) Measurement of static anterior tibial translation under monopodal weight‐bearing conditions, defined as the horizontal distance between Points B and C [4, 7].

An increased posterior tibial slope (PTS) influences anterior tibial translation under axial load (static anterior tibial translation, sATT) by increasing anterior shear forces [1, 2, 5, 14, 18, 23, 50]. Its clinical relevance lies not only in the measured angle but also in its functional manifestation. This translational behaviour is not determined by PTS alone [5, 13, 34, 54]. The medial meniscus, especially the posterior horn and ramp region, plays a critical secondary stabilizing role and may amplify slope‐driven sATT [5, 6, 13, 27]. TDO, therefore, addresses a structural sagittal‐plane problem. Its objective is to decrease sATT and reduce graft loading in knees where this mechanism is dominant [1, 2, 4, 23] (Figure 1).

LEAP, in contrast, is primarily designed to control rotational laxity [8, 16, 19, 22, 24, 45]. Over the past decade, it has consistently demonstrated the ability to reduce graft failure, particularly in young, pivoting athletes [11, 41, 42, 53]. Beyond rotational control, LEAP has been associated with lower global failure rates, fewer reinterventions and improved protection of repaired meniscus [31, 35, 36, 41, 42, 43]. In the majority of primary ACL reconstructions, especially when slope is within normal or mildly elevated ranges, LEAP represents a reproducible strategy to attenuate failure risk [3, 8, 11].

## LIMITS OF LEAP IN SLOPE‐DRIVEN INSTABILITY

While their protective role of LEAP in reducing graft failure is well established across different PTS values, this benefit appears to be progressively attenuated as the PTS increases [11]. In knees with markedly elevated PTS, the residual risk of graft failure may remain clinically relevant despite the addition of LEAP.

In a recent cohort of patients undergoing ACL reconstruction combined with LEAP, a PTS ≥12° was associated with graft rupture rates approaching 20% [23]. This risk increased further when combined with increased sATT, highlighting that the mechanical environment remains only partially addressed [23].

A subsequent analysis further demonstrated that this protective effect follows a nonlinear pattern, with LET performing optimally within a moderate slope range (5°–9°), while beyond 11°–12°, predicted failure probabilities rose sharply despite LET—reaching levels that may no longer be clinically acceptable [33].

**Figure 2:**
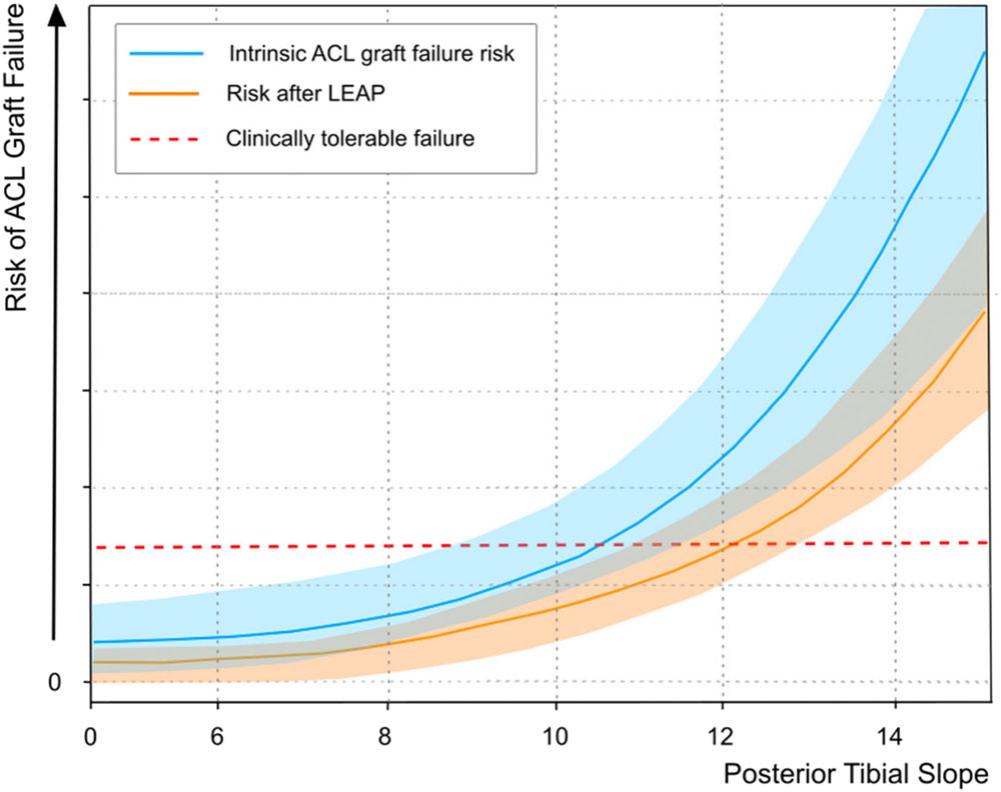
Conceptual representation of graft failure risk across increasing posterior tibial slope values. The blue curve and shaded area illustrate the baseline risk and its variability across patients, while the orange curve represents the relative reduction in risk associated with lateral extra‐articular procedures (LEAPs). The red dashed line denotes a clinically acceptable failure threshold. As slope increases, the protective effect of LEAP may be insufficient to maintain failure risk below clinically acceptable levels, highlighting the limitations of isolated rotational control in slope‐driven instability. ACL, anterior cruciate ligament.

In this context, the limitation is not the effectiveness of LEAP itself, but the persistence of sagittal‐plane laxity that remains uncorrected. These findings suggest that, in selected high‐risk phenotypes, rotational control alone may be insufficient to create a biomechanically favourable environment for graft survival [26, 32] (Figure 2).

**Figure 3:**
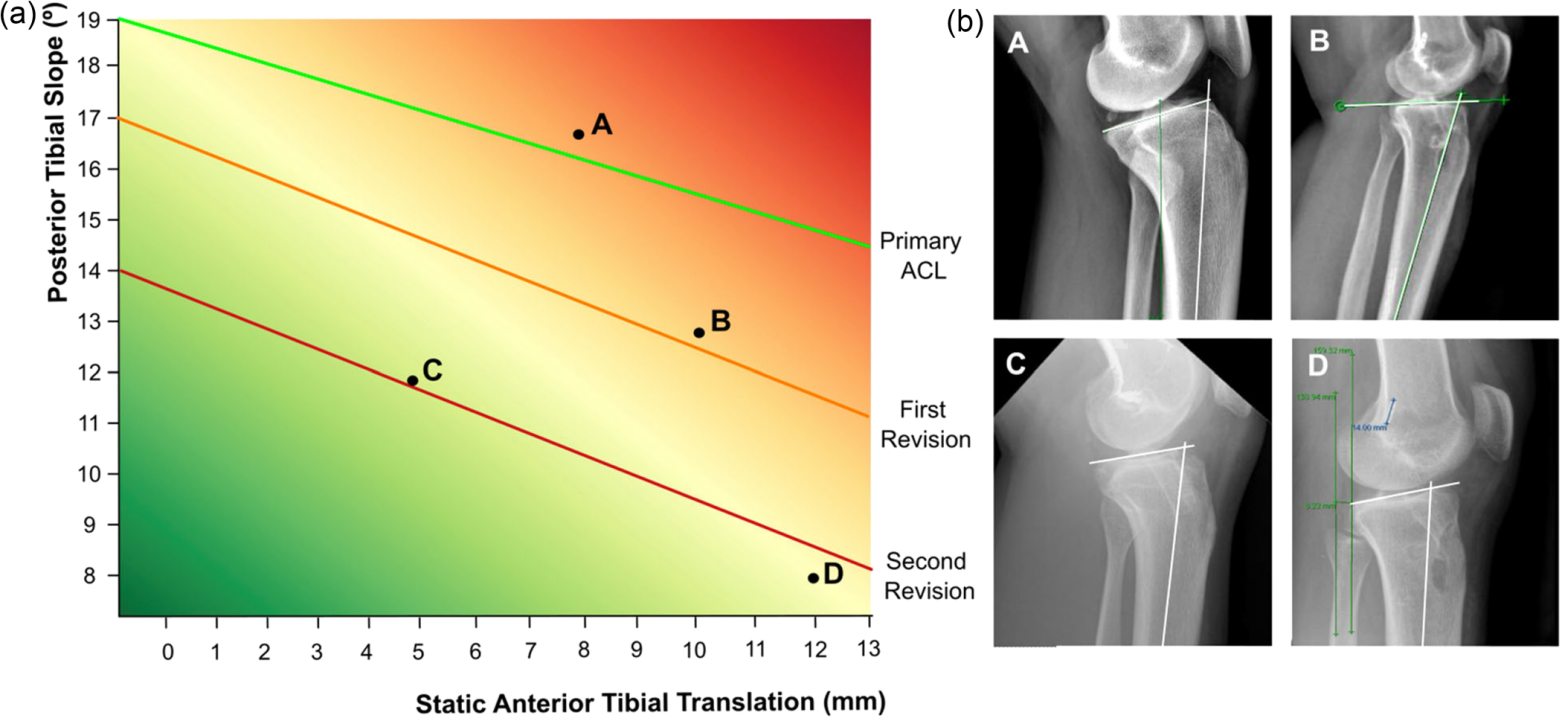
(a) Conceptual framework illustrating the interaction between posterior tibial slope (PTS) and static anterior tibial translation (sATT). The gradient represents increasing graft failure risk, and oblique lines indicate schematic thresholds for considering tibial deflexion osteotomy (TDO) according to surgical history. Points A–D correspond to the clinical cases shown in Panel (b). (b) Four illustrative clinical cases demonstrating different combinations of PTS and sATT: (a) 17°, 8 mm; (b) 13°, 10 mm; (c) 11°, 5 mm and (d) 12°, 8 mm. In all cases, TDO was recommended. ACL, anterior cruciate ligament.

The interpretation of this relationship must also consider that sagittal instability is not exclusively determined by PTS. Although PTS represents the principal structural contributor, sATT is influenced by multiple factors, including meniscal integrity, particularly of the medial posterior horn and ramp region, generalized ligamentous laxity and the chronicity of the lesion [5, 6, 10]. These elements may modulate both the magnitude and the clinical expression of translational instability, helping to explain why similar slope values can be associated with different clinical behaviours [4] (Figure 3).

## MATCHING THE PROCEDURE TO THE DOMINANT INSTABILITY DRIVER

The key is to determine which component of laxity predominates. Rotational laxity remains fundamentally a clinical diagnosis. A clear pivot shift represents dynamic rotational insufficiency and provides strong justification for LEAP [35]. Yet its grading is operator‐dependent and not always unequivocal. In borderline cases, adjunctive elements may assist interpretation [51]. These instruments do not replace clinical judgement but can provide additional clarity when the rotational phenotype is uncertain.

Sagittal‐plane laxity, by contrast, is less reliably captured by standard clinical testing. Lachman and anterior drawer examinations, as well as instrumented stress radiographs obtained without axial loading, frequently normalize after isolated ACL reconstruction. This behaviour is best appreciated on a lateral radiograph obtained in monopodal stance, where physiologic load is applied across the joint [2]. In this setting, persistent sATT may be evident despite apparently satisfactory passive laxity. While isolated ACL reconstruction can restore anterior control in non–weight‐bearing conditions, only TDO has consistently demonstrated attenuation of the repetitive shear forces generated by excessive slope under load [4, 32].

Whether monopodal weight‐bearing radiographs should be obtained routinely in all ACL patients remains debated. There is no universal consensus, particularly given that TDOs are uncommon in primary reconstruction [49]. However, selective use of weight‐bearing lateral imaging may provide valuable risk stratification [46]. Similarly, in the absence of weight‐bearing radiographs, anterior tibial translation may be suspected on MRI by assessing tibial position relative to the femur, using the PCL as an indirect reference [29, 39, 48]. Even if TDO is ultimately not indicated, identifying a translational phenotype enables more informed surgical planning and a more transparent discussion with the patient about failure risk [11, 23, 40]. Once these mechanisms are distinguished, surgical decision‐making becomes more coherent.

This distinction gains further importance in the revision setting. When graft failure occurs despite a prior LEAP procedure, simply repeating the anterolateral procedure without reassessing sagittal‐plane mechanics risks overlooking the primary mechanical driver [15, 30, 36]. In selected cases, persistent sATT attributable to excessive PTS may represent the uncorrected substrate underlying repeated failure.

The temporal evolution of the injury should also be considered. In the setting of an acute ACL rupture combined with increased PTS, measurable sATT may not yet be evident. In such cases, the absence of sATT changes should be interpreted with caution, as it may reflect the early stage of injury rather than a benign mechanical environment. Under axial load, repetitive shear forces may still be present, suggesting that slope‐related risk may exist even before overt translational patterns become established.

**Figure 4:**
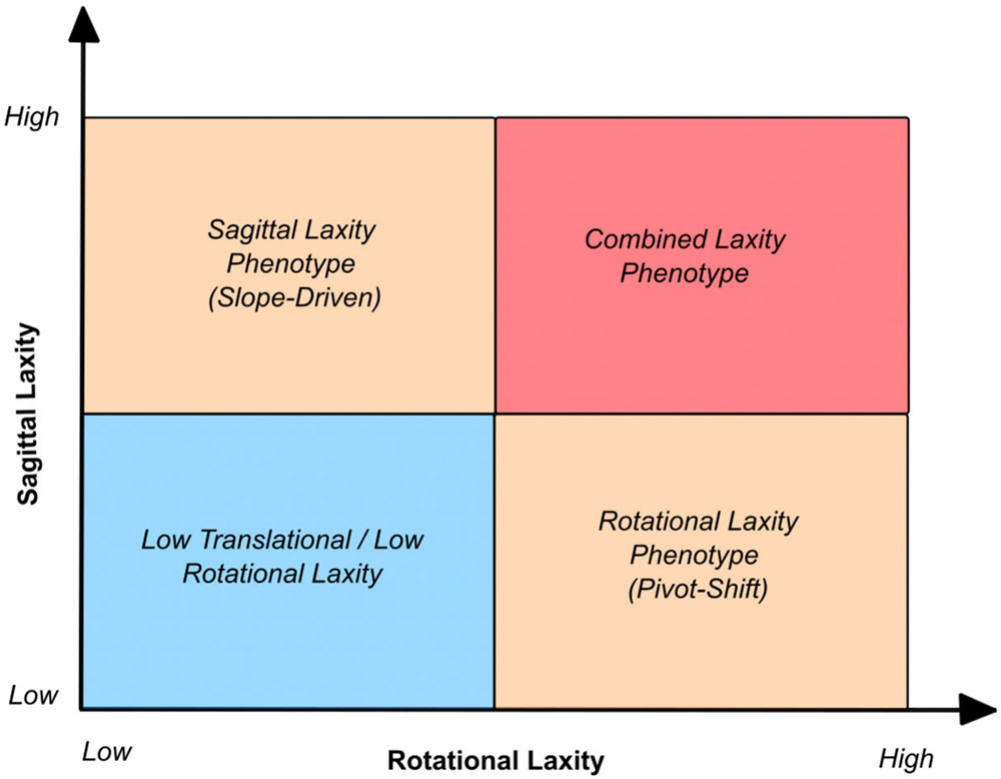
Conceptual classification of ACL instability based on sagittal (static anterior tibial translation) and rotational laxity, defining four phenotypes: low laxity, rotational, sagittal (slope‐driven) and combined. These phenotypes should be interpreted in conjunction with the cumulative patient‐specific risk factors for graft failure, rather than in isolation, to guide surgical decision‐making. ACL, anterior cruciate ligament.

In clinical practice, these laxity phenotypes rarely exist in isolation. Many patients present with elements of both sagittal and rotational laxity, forming a combined phenotype, while others may not exhibit a clearly predominant pattern. In such cases, laxity patterns must be interpreted in conjunction with patient‐specific risk factors for graft failure, such as age, activity level, generalized laxity, meniscal status and bony morphology, rather than as isolated constructs. This integrated approach allows surgical strategy to be tailored not only to the dominant mechanical driver, but to the overall risk profile of the patient (Figure 4).

In this context, risk factors should be interpreted cumulatively rather than in isolation [28]. While PTS alone may not mandate correction in all cases, its contribution within a high‐risk profile may justify a more proactive strategy. In selected patients, particularly young, high‐demand individuals presenting with multiple risk factors for graft failure, such as increased PTS, elevated static sATT, generalized laxity or meniscal tear, the threshold for addressing slope‐related mechanics may reasonably be lowered. In such scenarios, consideration of TDO during primary reconstruction may be appropriate as part of an individualized, risk‐adapted approach.

Finally, the magnitude of slope correction similarly requires individualized judgement. Risk associated with PTS increases progressively rather than abruptly [9, 37, 44]. In knees with moderate elevation and limited translational expression, incremental reduction may be biomechanically sufficient. Tailoring correction to the observed mechanical pattern reflects a shift away from rigid numeric targets toward functional alignment [25, 28, 38].

## WHO TRULY BENEFITS FROM ADDITIONAL PROCEDURES

Improved understanding of reinjury risk has also influenced the role of isolated ACL reconstruction. While isolated reconstruction remains effective in selected low‐risk patients, contemporary evidence suggests that additional stabilization may provide meaningful protective benefit in many active individuals [31, 42, 43]. The space for truly isolated ACL reconstruction may therefore be narrowing—not because it is obsolete, but because risk stratification has improved and adjunctive procedures have demonstrated additive value [35]. In this context, LEAP offers a favourable balance between protective benefit and procedural burden, with low rates of major complications [52]. Functional outcomes further support this approach, as return‐to‐sport rates remain high, particularly in young athletes returning to pivoting activities [17, 19].

In contrast, TDO should remain selective. In primary ACL reconstruction, most knees do not exhibit severe slope‐driven translational instability. Although elevated PTS is not uncommon, its presence alone does not mandate structural correction [28, 38]. Only when excessive slope is coupled with demonstrable sATT, and often additional factors such as meniscal deficiency, does slope correction become biomechanically compelling. In these infrequent scenarios, the structural contribution of slope may be decisive [47]. Yet this benefit must be weighed against the greater operative burden of an osteotomy and its distinct complication spectrum, most commonly related to hardware irritation and potential reintervention, even if severe adverse events remain uncommon [20]. Return‐to‐sport outcomes after combined ACL reconstruction and slope correction are encouraging, including reports of high‐level athletes resuming competition, but these cases typically reflect carefully selected, high‐risk phenotypes rather than routine primary reconstruction [21].

These strategies are not mutually exclusive [12]. The key question is whether the patient truly requires additional intervention and, if so, which mechanism is driving the risk of graft failure. In selected high‐risk knees, particularly in revision settings, addressing both rotational and sagittal components may be appropriate. Conversely, in the absence of a clearly defined mechanical driver, additional procedures may offer limited incremental benefit despite theoretical risk factors. Surgical decision‐making should therefore be guided by the relative contribution of each component of pathological laxity, ensuring that each intervention meaningfully addresses the underlying source of graft stress.

## CONCLUSION

The expanding use of LEAPs and slope‐reducing osteotomy reflects meaningful progress in our understanding of ACL failure mechanisms. Yet progress in technique must be matched by progress in indication.

Rotational laxity and structural anterior tibial translation are not interchangeable phenomena. When we fail to distinguish between them, we risk repeating procedures that do not address the dominant biomechanical driver—or introducing structural corrections to the bone where proportional soft‐tissue augmentation would suffice.

The future of ACL surgery does not lie in expanding indications indiscriminately, nor in favouring one strategy over another. It lies in defining instability phenotypes with clarity and aligning intervention with mechanism. Precision in diagnosis must precede precision in execution. Only then can surgical innovation remain both effective and proportionate.

## CONFLICT OF INTEREST STATEMENT

The authors declare no conflict of interest.

## ETHICS STATEMENT

The authors have nothing to report.
